# Left atrial volume predicts adverse cardiac and cerebrovascular events in patients with hypertrophic cardiomyopathy

**DOI:** 10.1186/1476-7120-9-34

**Published:** 2011-11-18

**Authors:** Tomoko Tani, Toshikazu Yagi, Takeshi Kitai, Kitae Kim, Hitomi Nakamura, Toshiko Konda, Yoko Fujii, Junichi Kawai, Atsushi Kobori, Natsuhiko Ehara, Makoto Kinoshita, Shuichiro Kaji, Atsushi Yamamuro, Shigefumi Morioka, Toru Kita, Yutaka Furukawa

**Affiliations:** 1 Department of Cardiovascular Medicine, Kobe City Medical Center General Hospital, 2-1-1 Minatojima-Minamimachi, Chuo-ku, Kobe 650-0047, Japan

**Keywords:** hypertrophic cardiomyopathy, left atrial volume, cardiac and cerebrovascular events, paroxysmal atrial fibrillation

## Abstract

**Aims:**

To prospectively evaluate the relationship between left atrial volume (LAV) and the risk of clinical events in patients with hypertrophic cardiomyopathy (HCM).

**Methods:**

We enrolled a total of 141 HCM patients with sinus rhythm and normal pump function, and 102 patients (73 men; mean age, 61 ± 13 years) who met inclusion criteria were followed for 30.8 ± 10.0 months. The patients were divided into two groups with or without major adverse cardiac and cerebrovascular events (MACCE), a composite of stroke, sudden death, and congestive heart failure. Detailed clinical and echocardiographic data were obtained.

**Results:**

MACCE occurred in 24 patients (18 strokes, 4 congestive heart failure and 2 sudden deaths). Maximum LAV, minimum LAV, and LAV index (LAVI) corrected for body surface area (BSA) were significantly greater in patients with MACCE than those without MACCE (maximum LAV: 64.3 ± 25.0 vs. 51.9 ± 16.0 ml, p = 0.005; minimum LAV: 33.9 ± 15.1 vs. 26.2 ± 10.9 ml, p = 0.008; LAVI: 40.1 ± 15.4 vs. 31.5 ± 8.7 ml/mm^2^, p = 0.0009), while there were no differences in the other echocardiographic parameters.

LAV/BSA of ≥ 40.4 ml/m^2 ^to identify patients with cardiovascular complications with a sensitivity of 73% and a specificity of 88%.

**Conclusion:**

LAVI may be an effective marker for detecting the risk of MACCE in patients with HCM and normal pump function.

## Introduction

Hypertrophic cardiomyopathy (HCM) is a complex primary and genetically transmitted cardiac disease with diverse clinical courses that include a stable clinical course, progressive congestive symptoms requiring therapeutic intervention, and sudden cardiac death [1,2]. Paroxysmal atrial fibrillation (PAF) is a common complication of HCM [3-5], often leading to acute or progressive heart failure and cerebral infarction. We previously showed that left atrial volume (LAV) was significantly greater in HCM patients with PAF than in those without PAF, and that LAV could more accurately predict the occurrence of PAF than could LA dimension [6]. Furthermore, it was recently shown that a dilated LA volume at baseline, a rapid increase in LA volume, and a New York Heart Association functional class were independent predictors of unfavorable outcomes in patients with HCM [7].

In the present study, we investigated the clinical significance of echocardiographic LAV, as well as other echocardiographic and clinical parameters, in HCM patients with preserved normal pump function. Furthermore, HCM patients were assessed for major adverse cardiac and cerebrovascular events (MACCE), and the relationship between LAV and MACCE was determined.

## Methods

### Study patients

We studied 141 consecutive patients diagnosed with HCM by two-dimensional echocardiography at our institution between January 2000 and February 2002. Exclusion criteria were chronic AF, other arrhythmias, prior stroke, permanent pacemaker implantation, and poor imaging. In this study, we excluded the patients with left ventricular (LV) outflow tract obstruction.

The final population consisted of 102 patients who met inclusion criteria. Although patients with hypertension were included in our study, the vast majority of hypertensive subjects carried only mild or well-controlled hypertension. All patients had normal pump function and were in sinus rhythm at the time of examination.

### Echocardiography

In each patient, the diagnosis of HCM was made on the basis of a two-dimensional echocardiographic finding of a hypertrophied and non-dilated LV (wall thickness ≧ 15 mm), in the absence of any cardiac or systemic disease capable of producing a comparable magnitude of LV hypertrophy. The greatest thickness measured at any site in the LV wall was considered to represent the maximal wall thickness. All patients except apical hypertrophy had asymmetric septal hypertrophy and septum/posterior wall thickness ratio of 1.3 or more. LAVs were assessed off-line using the modified biplane area-length method. LAV was determined at the time of mitral valve opening (maximal volume) and mitral valve closure (minimal volume). The area of the left atrium was determined by tracing the endocardial border of the left atrium in apical four-chamber and apical long-axis views. The length of the left atrium was measured from the midline of the mitral annulus to the opposite wall. Both the absolute volume (LAV) and the maximum volume indexed to body surface area (BSA) (LAV/BSA) were determined. LAV data were measured by an independent observer blinded to the groups.

Other echocardiographic parameters were obtained from the echocardiography reports. We retrieved LA diameter, LV end-systolic and end-diastolic dimensions, LV septal and posterior wall thickness, LV ejection fraction (EF), peak mitral flow velocity of the early rapid filling wave (E), peak velocity of the late filling wave due to atrial contraction (A), the E/A ratio, and the deceleration time of the early mitral filling wave (DcT). LA diameter was measured in a parasternal long-axis view. LA diameter and LV wall thickness were obtained according to the standards of the American Society of Echocardiography (ASE) [8]. EF was calculated from apical two-and four-chamber views according to the biplane modified Simpson rule. The cut-off used for normality of EF was 60%.

The severity of mitral regurgitation (MR) was semiquantitatively evaluated by color Doppler echocardiography in the apical four-chamber view. We measured MR jet area and LA area and calculated the ratio of MR jet area to LA area. They were graded as none, mild (ratio jet area/LA area <20%), and moderate or severe (jet area/LA area > 20%).

### Identification of PAF

Clinical information was obtained from medical records. All patients were followed up in our hospital. The presence of PAF was documented by either 12-lead resting electrocardiogram (ECG) or 24 h ambulatory ECG. The patients with PAF had at least one symptomatic episode. The patients with sinus rhythm were free from chest symptoms and documentation of PAF using the above criteria. No patients were complicated with congestive heart failure or other arrhythmias at the time of examination.

### Follow-up data

We followed up the study patients for a follow-up of 30.8 ± 10.0 months (median, 31 months; longest observation, 80 months). The primary endpoints were major adverse cardiac and cerebrovascular events (MACCE), defined as hospitalization for worsening congestive heart failure, a composite of stroke and sudden cardiac death. Sudden cardiac death was defined as unexpected sudden cardiac collapse within one hour of new symptoms or nocturnal death with no antecedent history of worsening of symptoms. MACCE data were obtained from charts or by telephone interview with the patients.

### Statistical analysis

All continuous variables are expressed as mean ± SD if the values were parametrically distributed. Values were compared between the groups by unpaired Student's *t *test as they were parametrically distributed. A *p*-value <0.05 was considered statistically significant. Receiver operating characteristics (ROC) curve analysis to determine the optimal cut-off values for the maximum LAV, and LAV/BSA was used to distinguish HCM patients at higher risk of MACCE. Survival curves were constructed according to the Kaplan-Meier method. Multivariate Cox regression analysis was performed to assess echocardiographic parameters for MACCE. All statistical analyses were performed using SPSS statistical software (Version 11; SPSS Inc., Chicago, IL, United States).

## Results

### Baseline characteristics

Of the 141 consecutive patients eligible for the present study, 102 patients (61 ± 13 years) were followed up for the occurrence of MACCE. The median follow-up period was 30.8 months (range, 12-80 months). MACCE occurred in 24 patients (Group A), while the remaining 78 patients showed no signs of MACCE (Group B). The baseline clinical characteristics of the study population are summarized in Table 1. The prevalence of hypertension and PAF was significantly higher in Group A than in Group B. There were no significant differences in any other clinical and laboratory variables between the two groups.

**Table 1:** Baseline clinical characteristics of the study population

	Total	Group A	Group B	P-value
Age	61 ± 13	64 ± 12	59 ± 12	0.14

Male	73 (72%)	18 (74%)	55 (71%)	0.63

BPs (mmHg)	130 ± 19	137 ± 23	128 ± 17	0.04

BPd (mmHg)	77 ± 10	79 ± 10	76 ± 10	0.14

Height (m)	1.62 ± 0.09	1.60 ± 0.08	1.63 ± 0.09	0.12

Weight (kg)	61.8 ± 11.1	61.9 ± 9.6	61.7 ± 11.8	0.94

BSA (m^2 ^)	1.67 ± 0.18	1.65 ± 0.16	1.67 ± 0.19	0.75

History of HT	43 (42%)	16 (67%)	27 (35%)	<0.0001

DM	10 (10%)	3 (13%)	7 (9%)	0.37

HL	49 (48%)	12 (50%)	37 (47%)	0.67

PAF	23 (23%)	13 (54%)	10 (13%)	<0.0001

Medications Aspirin	15 (15%)	8 (33%)	7 (9%)	<0.0001

Warfarin	4 (17%)	4 (17%)	0	<0.001

Calcium ant.	41 (40%)	11 (46%)	30 (38%)	0.25

Beta-blockers	44 (43%)	11 (46%)	33 (42%)	0.57

Diuretics	6 (6%)	3 (13%)	3 (4%)	0.02

ACE inhibitor	19 (19%)	8 (33%)	11 (14%)	0.002

BPs, systolic blood pressure; BPd, diastolic blood pressure

BSA, body surface area; PAF, paroxysmal atrial fibrillation

In Group A, during follow-up, there were 24 events of MACCE: 18 strokes, 4 congestive heart failure and 2 sudden deaths (Table 2). In 18 patients presented with stroke, the rhythm of 7 patients was atrial fibrillation at the time of complications.

**Table 2:** Events of 24 patients with Hypertrophic Cardiomyopathy

Events	N = 24
stoke	18 (75%)

congestive heart failure	4 (17%)

sudden death	2 (8%)

### Morphologic classification of HCM

The study patients included 21 patients with apical hypertrophy, seven patients with basal hypertrophy, four patients with diffuse hypertrophy of the ventricular septum, 61 patients with diffuse hypertrophy of the ventricular septum and the antero-lateral free wall, and nine patients with hypertrophy of the lateral wall (Table 3).

**Table 3:** Morphologic classification of HCM

	Total	Group A	Group B	p-value
APH	21 (21%)	6 (25%)	15 (19%)	0.31

Type 1	7 (7%)	1 (4%)	6 (8%)	0.23

Type 2	4 (4%)	1 (4%)	3 (4%)	1.00

Type 3	61 (60%)	14 (59%)	47 (60%)	0.89

Type 4	9 (9%)	2 (8%)	7 (9%)	0.80

APH, apical hypertrophy

Type 1, basal septal hypertrophy

Type 2, diffuse hypertrophy of the ventricular septum

Type 3, diffuse hypertrophy of the ventricular septum and antero-lateral free wall

Type 4, hypertrophy of the lateral wall

### LV function

All patients had normal pump function. LV end-diastolic dimension, LV end-systolic dimension, interventricular septal wall thickness, and EF were not significantly different between Group A and Group B (Table 4). LV diastolic functions are also shown in Table 4, and there were no differences between the two groups.

**Table 4:** Baseline echocardiographic characteristics of the study population

	Total	Group A	Group B	p-value
LAD (cm)	3.81 ± 0.60	3.91 ± 0.56	3.80 ± 0.59	0.40

LAV, max (ml)	54.6 ± 19.2	64.3 ± 25.0	51.9 ± 16.0	0.005

LAV, min (ml)	27.9 ± 12.4	33.9 ± 15.1	26.2 ± 10.9	0.008

LAV/BSA (ml/m^2^)	34.0 ± 11.8	40.1 ± 15.4	31.5 ± 8.7	0.0009

LVD, diastole (cm)	4.34 ± 0.89	4.36 ± 0.51	4.33 ± 0.49	0.77

LVD, systole (cm)	2.59 ± 0.54	2.64 ± 0.50	2.57 ± 0.55	0.62

IVS (cm)	1.56 ± 0.43	1.59 ± 0.45	1.50 ± 0.40	0.37

EF (%)	66.8 ± 6.6	67.5 ± 6.2	66.5 ± 6.8	0.53

E/A	1.04 ± 0.4	1.2 ± 0.4	1.0 ± 0.4	0.42

DcT (msec)	244 ± 79	243 ± 71	254 ± 103	0.57

MR None	15 (15%)	2 (8%)	13 (17%)	0.05

Mild	77 (76%)	18 (75%)	59 (76%)	0.87

Moderate or severe	10 (10%)	4 (17%)	6 (7%)	0.03

LAD, left atrial diameter; LAV, left atrial volume

BSA, body surface area; LVD, left ventricular dimension

IVS, intraventricular septum; EF, ejection fraction

E/A, ratio of early to late transmitral peak flow velocity

DcT, deceleration time; MR, mitral regurgitation

### Incidence and severity of MR

Only 10 patients had moderate or severe MR. In Group A, four patients (17%) had moderate or severe MR. The prevalence and the severity of MR were significantly greater in Group A than in Group B (Table 4).

### LA size

**Figure 1:**
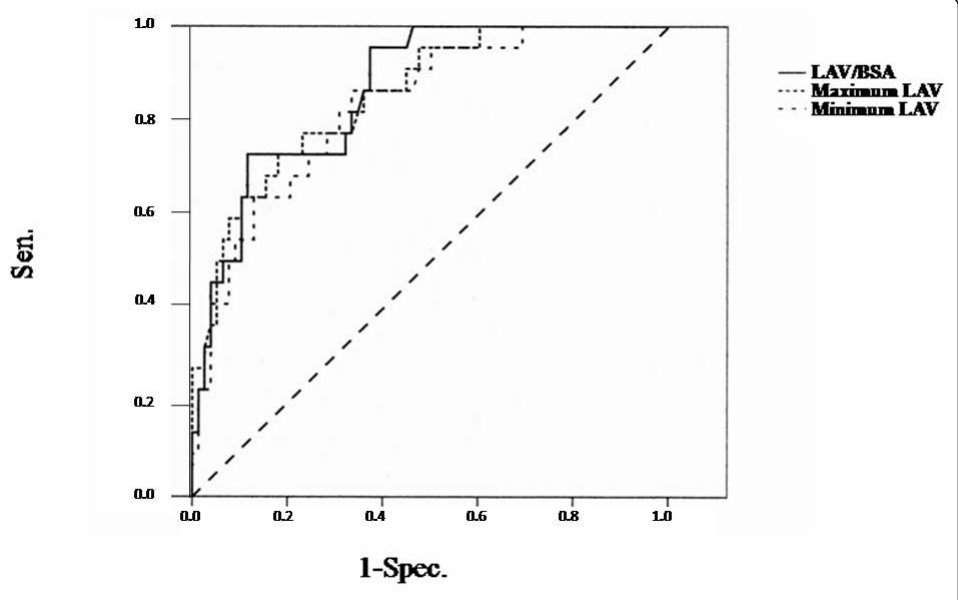
**Receiver operating characteristics curve for determining the optimal cut-off value for identifying patients with cardiovascular complications from maximum left atrial volume (LAV), minimum LAV and LAV corrected to body surface area (LAV/BSA); *Sen*., sensitivity; *Spec*., specificity**.

Although there were no significant differences in LA diameter between the two groups maximum LAV, minimum LAV, and LAV/BSA were significantly greater in Group A than in Group B (Table 4). ROC curve analysis revealed a maximum LAV of ≥ 62.7 ml to identify patients with cardiovascular complications with a sensitivity of 73% and a specificity of 82% (area under ROC curve = 0.853). ROC curve analysis revealed a LAV/BSA of ≥ 40.4 ml/m^2 ^to identify patients with cardiovascular complications with a sensitivity of 73% and a specificity of 88% (area under ROC curve = 0.864) (Figure 1).

**Figure 2:**
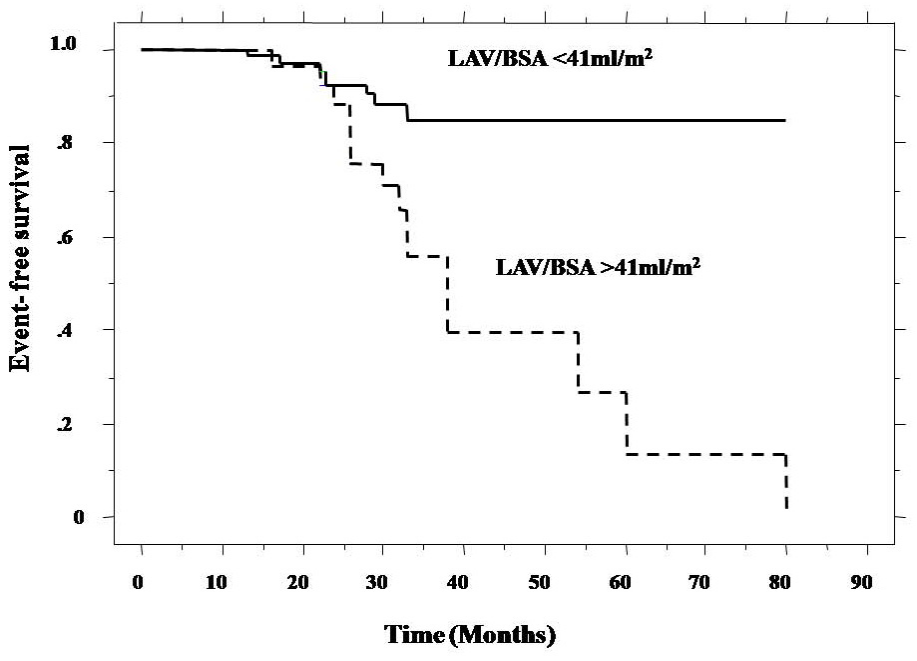
**Kaplan-Meier curves for MACCE, with the log-rank test (p = 0.001)**.

The Kaplan-Meier analysis also showed poor outcomes for MACCE in patients with LAV/BSA > 41 ml/m^2 ^(p = 0.001, Log-rank test) (Figure 2).

From our study, there were no patients with MACCE in the group with a LAV/BSA < 41 ml/m^2 ^after 33 months. By multivariate Cox regression analysis, LAV/BSA was an independent predictor for MACCE (Table 5).

**Table 5:** Predictors of MACCE by Cox regression multivariable analysis

	RR(95% CI)	Significance
LAD (cm)	0.86 (0.22-3.36)	0.83

LAV, max (ml)	1.03 (0.95-1.11)	0.51

LAV, min (ml)	0.98 (0.90-1.06)	0.57

LAV/BSA (ml/m^2^)	0.88 (0.80-0.97)	0.009

LAD, left atrial diameter; LAV, left atrial volume

BSA, body surface area;

## Discussion

In the present study, LA volume assessed by echocardiography was strongly associated with future risk of MACCE (a composite of stroke, sudden death, and congestive heart failure) in HCM patients with normal pump function. However, LA diameter, a standard measure used to assess LA size, was not associated with the risk of MACCE. A previous study reported that HCM patients have a 4-6-fold increased risk of developing AF compared with the general population [5]. Furthermore, once AF has developed the risk of ischemic stroke may increase 8-fold in HCM patients relative to HCM patients with sinus rhythm, presumably via an increase in cardioembolic stroke [9]. In our study the risk of stroke was comparable between patients with chronic AF and PAF.

### Outcome and LA volume

We previously reported that LA volume in sinus rhythm may be a strong predictor of future AF in patients with HCM [6]. Mild LA enlargement is common in HCM, and may be a consequence of impaired diastolic function associated with the thickened and noncompliant LV [10. 11]. Although assessment of LA enlargement appears to provide important information about the patients' outcome, unidimensional M-mode LA diameter cannot accurately measure LA size [12]. Similar to that previously described, our series indicated that LA volume was a better predictor of PAF and MACCE than was superior LA diameter [6,13-15].

### AF and stroke

LA dilatation promotes stasis of blood, which in turn predisposes to thrombus formation and the potential for embolization. In our study, LA size was associated with important risk factors for stroke and death. These observations underline the benefits of defining clinical markers able to non-invasively identify those patients with HCM who are at risk of developing PAF and suffering complication.

There are several factors that may influence outcome in patients with HCM and the occurrence of stroke and LA enlargement.

Indeed, in this study, Group A had a significantly greater number of patients with hypertension and PAF both of which may predispose to stroke and heart failure.

MR influences also in a relevant fashion LA volumes. In our study, Group A had a significantly greater number of patients with severe MR.

### Echocardiography and risk for sudden death assessment

Using two-dimensional echocardiographic measurements, Spirito et al. showed that the risk of sudden death increased progressively in direct relation to wall thickness [3]. But, there were no significant differences of the distribution of hypertrophy between two groups in our study.

Previous paper showed that LV outflow tract obstruction is associated with an increased risk of sudden death [16]. Otherwise, Losi MA et al. showed that there was no statistical differences between patients with and without obstruction on outcome [7].

Clinically, HCM patients without obstruction were sometimes occurred complications. There were some clinical significances of echocardiographic parameters in patients with nonobstructive hypertrophic cardiomyopathy.

### Study limitations

First, the method we used for detection of PAF based on clinical documentation with electrocardiographic confirmation may have underestimated the actual incidence of PAF. Some patients with AF may have been asymptomatic, and if they did not present to the hospital then the arrhythmia could have remained undetected. However, all patients presented at least once to our hospital within a 1-year period. 7 of 18 patients were in AF at the time they presented with a stroke. Second, we only measured the LA volume at baseline, not during follow-up. As the dilatation rate of LA volume may also be an important prognostic factor in patients with HCM. And in our study, we did not investigate the patients with LV outflow obstruction. Previous paper showed that LV outflow tract obstruction is associated with an increased risk of sudden death [16]. In this study, we excluded the patients with obstruction. We investigated whether LA volume predict adverse cardiac and cerebrovascular events in HCM patients, as exclusive for the influence of LV outflow tract obstruction. Finally, diastolic dysfunction is determined with deceleration time of LV inflow, occult systolic dysfunction detected with tissue Doppler analysis. We did not use tissue Doppler imaging in our study.

Nistri s et al. reported that body mass index and the ratio of early diastolic peak LV inflow velocity to peak myocardial early diastolic velocity (E/e') ratio affect LAV/BSA only in non-athletes. Many factors may influence LA volume [17].

## Conclusion

LAV indices are sensitive markers for detecting the risk of MACCE in patients with HCM who show normal pump function.

## List of abbreviations

HCM: hypertrophic cardiomyopathy; PAF: paroxysmal atrial fibrillation; LAV: left atrial volume; MACCE: major adverse cardiac and cerebrovascular events; LV: left ventricular; BSA: body surface area; EF: ejection fraction; MR: mitral regurgitation; ECG: electrocardiogram.

## Competing interests

The authors declare that they have no competing interests.

## Authors' contributions

TY, HN, TK, YF and JK performed echocardiography.

All authors read and approved the final manuscript.
